# De-differentiated giant thigh liposarcoma disguised as recurrent lipoma; a case report

**DOI:** 10.1016/j.ijscr.2022.107292

**Published:** 2022-06-07

**Authors:** Nebiyou Simegnew Bayileyegn, Amare Abera Tareke

**Affiliations:** a Department of Surgery, Jimma University Medical Center, Jimma, Ethiopia; b Department of Biomedical Sciences, College of Medicine and Health Sciences, Wollo University, Dessie, Ethiopia

**Keywords:** Liposarcoma, Recurrent, Dedifferentiated liposarcoma, Sarcoma, Radiotherapy

## Abstract

**Introduction and importance:**

Lipomas are one of the commonest soft tissue tumors which usually arise from subcutaneous plane. Lipomas can progress to one of the three types of liposarcomas. Dedifferentiated liposarcomas have metastasis potential, risk of recurrence and increased risk of mortality. The culture of taking every lipoma as benign should be seriously considered with different approaches as it can lead to sarcomatous degeneration in some cases.

**Case presentation:**

A 78 years old male patient present to our hospital with recurrent left thigh swelling of two years duration with limping and sense of heaviness on left side. He has history of previous surgery for the same complaint on the same site two times, six and four years ago. Histological diagnosis in both cases was lipoma. Objectively he has 20 × 60 cm non tender, soft to firm, slightly mobile swelling on left thigh spanning anterior, lateral and posterior compartment but no inguinal swelling. Fine needle aspiration cytology from the mass reported sarcoma. CT-scan stated diagnosis as sarcomatous tumor.

**Clinical discussion:**

Lipoma is commonest soft tissue tumor arising from adipocytes more often in young adults, women and overweight population. Well-differentiated liposarcoma can progress to dedifferentiated liposarcoma over time which is slightly aggressive than its precursor. Management of liposarcoma depends on stage, site and histology. Surgical resection with radiotherapy is the standard of care for localized dedifferentiated liposarcomas.

**Conclusion:**

It is difficult to differentiate between giant lipoma and well-differentiated liposarcoma with physical examination and imaging. Histopathologic examination in uncertain conditions will decrease morbidity and mortality from the more aggressive forms of liposarcomas.

## Highlights

• Lipoma is one of the commonest soft tissue tumors which usually arise from subcutaneous plane.
• Well- differentiated liposarcomas more resembles lipoma except for its enlarged nuclei and some degree of atypia.
• Dedifferentiated liposarcoma has metastasis potential, risk of recurrence and increased risk of mortality.
• Surgical resection with radiotherapy is the standard of care for localized dedifferentiated liposarcomas.

## 1 Introduction

Lipomas are one of the commonest soft tissue tumors which usually arise from subcutaneous plane. As it arises from fat cells, it can be found in any plane/part in the body [1]. Large or giant lipoma is considered when the size is more than 5 cm. Differentiation between lipoma and low malignant potential liposarcoma is a diagnostic challenge both consisting of single or multiple fat globules and therapeutic management is different [1], [2].

Three main types of liposarcoma include well-differentiated/dedifferentiated, myxoid/round cell and pleomorphic. Well-differentiated and dedifferentiated liposarcomas are most common types and share the same genetic alteration [3]. Well- differentiated liposarcomas more resembles lipoma except for its enlarged nuclei and some degree of atypia and it can progress to de-differentiated liposarcoma [4], [5]. Unlike well-differentiated liposarcoma, dedifferentiated, liposarcoma has metastasis potential, risk of recurrence and increased risk of mortality at five years [6]. We present the history and management of massive dedifferentiated left thigh liposarcoma which has been previously resected, and disguised as recurrent lipoma. Case reporting was done according to SCARE 2020 guideline [7].

## 2 Case presentation

A 78 years old male patient presents to our hospital with recurrent left thigh swelling of two years duration with limping and sense of heaviness on left side. He has history of previous surgery for the same complaint on the same site two times, six and four years ago. The diagnosis which was confirmed histologically after both resections was lipoma. There was no further treatment after resection. The patient has no other site swelling, cough, bone pain or abdominal pain. The patient has no other history of chronic illness.

**Fig. 1:**
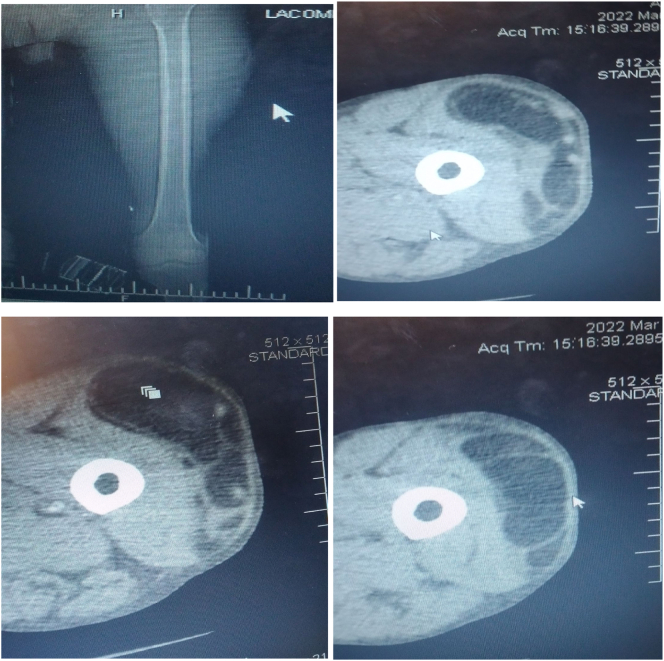
A-D: Cross-sectional CT-images of the mass demonstrating hypodense lesion with heterogenous, irregular and septated lesion.

Objectively, he was comfortable with stable vital signs. There was no lymphadenopathy, chest was clear and resonant. There are two previous incision sites on left thigh. There was 20 × 60 cm non tender, soft to firm, slightly mobile swelling on left thigh spanning anterior, lateral and posterior compartment, no inguinal swelling. All blood work (complete blood count and organ function tests) were normal. Fine needle aspiration cytology from mass reported spindle cell tumor with mixed mesenchymal and fatty cells. Chest X-ray and abdominal ultrasound for metastasis were negative. CT-scan of chest, abdomen and pelvis for metastasis were free. CT-scan of the involved extremity showed 20 × 50 × 20 cm left thigh soft tissue mass involving anterior, lateral and posterior compartment extending from supra-knee area to mid of femoral triangle and up to gluteal region (10 cm below iliac crest) postero-laterally. There is no involvement of femoral vessels and there is no inguinal mass. Fine needle aspiration cytology from the mass reported sarcoma. CT-scan stated diagnosis as sarcomatous tumor (Fig. 1A-D).

**Fig. 2:**
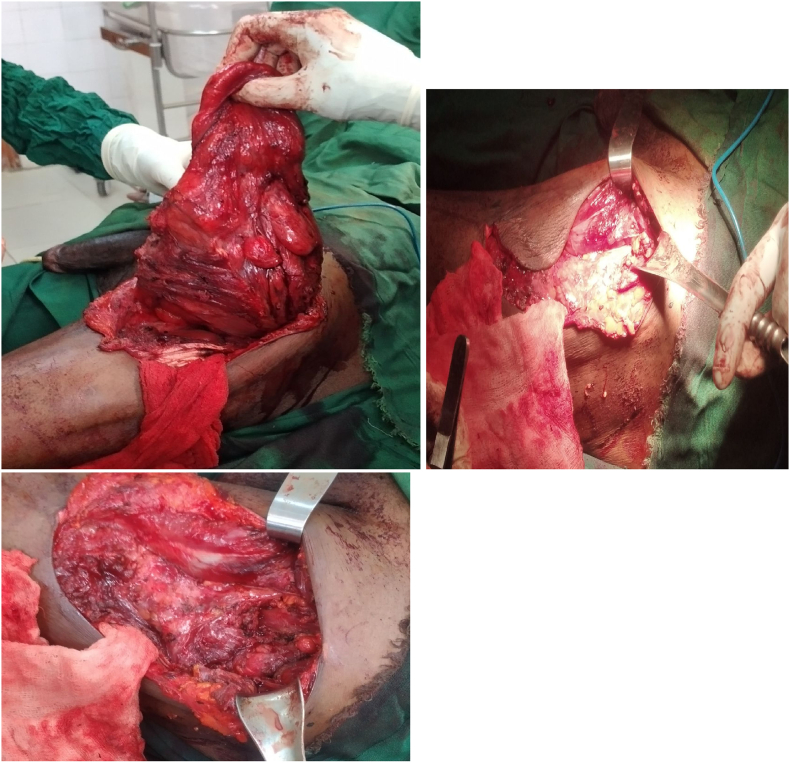
A-C: Intraoperative images showing the tumor and post-excisional dead space.

**Fig. 3:**
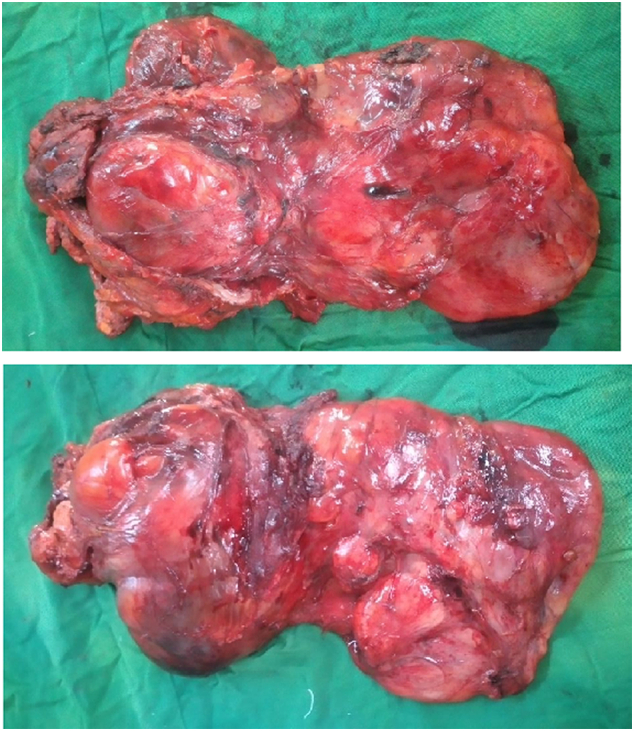
A & B: post-excisional images of the tumor.

Patient was prepared for operation with informed consent and prophylaxis antibiotics. We made Lazy-S incision over the mass. There was 20 × 60 cm mass soft to firm consistency, mostly of fatty appearance and firm on superior aspect. The mass adheres to femoral sheath and infiltrates some part of sartorius and vastus lateralis muscles but no neurovascular invasion (Fig. 2A-C). Wide local excision with macroscopic negative margin was done and the big dead space closed with approximation of muscle (Fig. 3A & B). Histopathologic exam of the specimen turned out to be dedifferentiated liposarcoma. Post-operative recovery was uneventful. On postoperative day 2, patient started to move on rolling walker support and on postoperative day 4, patient started on flexion and extension of hip and knee joint with slight discomfort. The patient was discharged on postoperative day 7 for radiotherapy. The patient completed his course of radiotherapy (50 Gy) and we reevaluated him after 2 months, the therapy was well tolerated and patient was able to walk with support of stick. He reported general feeling of wellbeing after the combined treatment.

## 3 Discussion and conclusion

Lipoma is commonest soft tissue tumor arising from adipocytes more often in young adults, women and overweight population. When the size enlarges to more than 5 cm, it alerts other malignant lipomatous tumor transformation [1]. In our case, a 73 years old male apparently healthy person, who had two previous resections for lipoma six and four years back respectively has this massive swelling over his thigh; which makes a fast transformation in a period of four years.

Well-differentiated liposarcoma can progress to dedifferentiated liposarcoma over time which is slightly aggressive than its precursor. In a study by Thirasastr and Somaiah, it was found that 10 % of liposarcoma arise as recurrence after resection of well-differentiated liposarcoma, while the rest 90 % progressed from undiagnosed well-differentiated liposarcoma [3]. In our case, it seems that, the tumor has progressed from missed well-differentiated liposarcoma, supported by evidence of previous surgical resections on the same site. Dedifferentiated liposarcoma is a high-grade aggressive tumor with locally infiltrative and distant metastasis potentials [8]. Dedifferentiated liposarcoma commonly arise in the extremities and retroperitoneum but rarely in the mediastinum, para-testicular region or spinal cord. Retroperitoneal liposarcomas diagnosed at later stage and they have increased risk of local recurrence and metastasis compared to those arising in the extremities [6]. Dedifferentiated liposarcoma has a male predilection with secondary dedifferentiation having less metastatic potential than the de novo dedifferentiated liposarcoma. Retroperitoneum and lungs are the common metastatic sites whenever it happens [9].

Management of liposarcoma depends on stage, site and histology. Surgical resection with radiotherapy is the standard of care for localized dedifferentiated liposarcomas. Multimodality approach for advanced diseases is amenable although prognosis is poor in this group of patients [3], [10]. High-grade dedifferentiated liposarcoma of the extremity primarily managed with surgical resection of the tumor with 1 cm margin whenever resection can be undertaken without major morbidity [11]. Whenever the tumor encases major arteries, resection and reconstruction will decrease local recurrence. One-centimeter normal tissue or resection with negative fascial compartment is strongly advised. Adjuvant radiotherapy is indicated for those with positive resection margin or tumors more than 5 cm in size. There is no need of chemotherapy in localized extremity liposarcomas [12], [13]. Radiotherapy is recommended for local control. Yang randomized 141 patients to receive adjuvant external-beam radiation or no radiation following limb-sparing surgery. At median follow up of 9.3 years, local recurrence was 24 % for patients receiving no radiation and 0 % for patients receiving adjuvant radiotherapy. Among the low-grade group, the majority of patients again had a tumor located in the proximal lower extremity. At median follow up of 9.9 years, local recurrence was 33 % for patients receiving no radiation, compared with 0 % for patients who received radiotherapy. These differences in local recurrence were statistically significant [14]. In our case, the tumor abuts to femoral sheath and infiltrates local muscle, we resected femoral sheath and involved muscle segment along with the tumor and biopsy confirmed negative resection margin. Since the tumor measures 20 × 60 cm, patient received adjuvant radiotherapy.

Management of advanced liposarcomas consists of systemic chemotherapy, radiotherapy and surgical resection whenever possible. Systemic therapies historically limited only to cytotoxic chemotherapeutic agents. Recent studies showed improved effects with combination of cytotoxic agents and tyrosine kinase inhibitors. Clinical trials showed target therapies with cyclin-dependent kinase 4 (CDK4) inhibitor *abemaciclib* and the nuclear export inhibitor *Selinexor* [15], [16].

It is difficult to differentiate between giant lipoma and well-differentiated liposarcoma with physical examination and imaging. Histopathologic examination in uncertain conditions and the above precursor cases will decrease morbidity and mortality from the more aggressive forms of liposarcomas.

## Consent

Written informed consent was obtained from the patient for publication of this case report and accompanying images. A copy of the written consent is available for review by the Editor-in-Chief of this journal on request.

## Provenance and peer review

Not commissioned, externally peer-review.

## Sources of funding

This work does not receive funds.

## Ethical approval

Not applicable.

## Author contribution

Nebiyou Simegnew contributed substantially from the patient evaluation to writing up, Amare Amare contributed in revision of the paper.

## Registration of research studies

1. Name of the registry: researchregistry.com.
2. Unique identifying number or registration ID: researchregistry7735.
3. Hyperlink to your specific registration (must be publicly accessible and will be checked): https://www.researchregistry.com/browse-the-registry#home/.

## Guarantor

Nebiyou Simegnew will take the primary responsibility of the study.

## Declaration of competing interest

The authors declare that they have no competing interests.
